# Depressed hydraulic redistribution of roots more by stem refilling than by nocturnal transpiration for *Populus euphratica* Oliv. in situ measurement

**DOI:** 10.1002/ece3.3875

**Published:** 2018-02-05

**Authors:** Tengfei Yu, Qi Feng, Jianhua Si, Patrick J. Mitchell, Michael A. Forster, Xiaoyou Zhang, Chunyan Zhao

**Affiliations:** ^1^ Alxa Desert Ecohydrology Experimental Research Station Northwest Institute of Eco‐Environment and Resources Chinese Academy of Sciences Lanzhou China; ^2^ Key Laboratory of Ecohydrology of Inland River Basin Chinese Academy of Sciences Lanzhou China; ^3^ CSIRO Land and Water Hobart TAS Australia; ^4^ Edaphic Scientific Pty Ltd Port Macquarie NSW Australia; ^5^ School of Agriculture and Food Science The University of Queensland Brisbane Australia

**Keywords:** desert riparian trees, hydraulic redistribution, nocturnal transpiration, sap flow, stem refilling

## Abstract

During the night, plant water loss can occur either through the roots, as hydraulic redistribution (HR), or through the leaves via the stoma, as nocturnal transpiration (*E*_n_), which was methodologically difficult to separate from stem refilling (*R*_e_). While HR and *E*_n_ have been reported across a range of species, ecosystem, and climate zone, there is little understanding on the interactions between *E*_n_ and/or *R*_e_ and HR. As water movement at night occurs via gradients of water potential, it is expected that during periods of high atmospheric vapor pressure deficit (VPD), water loss via *E*_n_ will override water loss via HR. To test this hypothesis, sap flow in stems and roots of *Populus euphratica* Oliv. trees, growing in a riparian zone in a hyperarid climate, was measured once in a year. Nocturnal stem sap flow was separated into *E*_n_ and *R*_e_ using the “forecasted refilling” method. Substantial nocturnal sap flow (38% of 24‐hr flux on average) was observed and positively correlated with VPD; however, the strength of the correlation was lower (*R* ^2^ = .55) than diurnal sap flow (*E*_d_) (*R* ^2^ = .72), suggesting that nocturnal stem sap flow was attributed to both water loss through the canopy and replenishment of water in stem tissues. Partitioning of nocturnal sap flow shows that *R*_e_ constituted approximately 80%, and *E*_n_ ~20%, of nocturnal sap flow. The amount of root sap flow attributed to redistribution was negatively related to *E*_d_ (*R* ^2^ = .69) and the amount of acropetally sap flow in stems, *R*_e_ (*R* ^2^ = .41) and *E*_n_ (*R* ^2^ = .14). It was suggested that the magnitude of HR is more strongly depressed by *R*_e_ that was recharge to the water loss via *E*_d_ than by *E*_n_. It was consistent with whole‐tree water balance theory, that the nighttime upward sap flow to xylem, stem refilling and transpiration, may depress hydraulic redistribution of roots.

## 1 INTRODUCTION

During the night, plant water movement can occur via two major pathways: (1) the roots, where the movement of water is from different parts of the root zone with large gradients in water status [hydraulic redistribution (HR)] (Burgess, Adams, Turner, & Ong, 1998; Nadezhdina et al., 2010) or (2) the leaves, nocturnal transpiration (*E*_n_) from the canopy via the stoma (Dawson et al., 2007; Howard, van Iersel, Richards, & Donovan, 2009). While HR and *E*_n_ have been reported across a range of species, ecosystem, and climatic conditions, the relationship between both of them remains poorly understood (Bauerle, Richards, Smart, & Eissenstat, 2008; Dawson et al., 2007; Howard et al., 2009). Thus, determining the patterns of HR and *E*_n_ and their interaction is important to better understand plant water relations and ecosystem water balance.

It has been widely documented that HR can occur between the roots and soil environment in upward, downward, and lateral directions (Burgess et al., 1998; Hultine, Scott, Cable, Goodrich, & Willams, 2004; Nadezhdina et al., 2010; Prieto, Armas, & Pugnaire, 2012; Richard & Caldwell, 1987; Smith, Jacson, Roberts, & Ong, 1999) across a broad range of species and ecosystem, but it is particularly prevalent in arid and semiarid ecosystems (Caldwell & Richard, 1989; Hultine, Cable, Burgess, & Williams, 2003; Hultine et al., 2004; Yoder & Nowak, 1999; Yu & D'Odorico, 2015; Yu et al., 2013). HR most often occurs at night when the soil water potential of layers is larger than the canopy water potential while transpiration is low (Burgess et al., 1998; Prieto et al., 2012). However, where stem sap flow is significant, the predominant flow of water may be toward the leaves and thus override the driving force of water flow between root compartments (Burgess & Bleby, 2006; Howard et al., 2009; Neumann et al., 2014).

Owing to the water potential gradient between leaves and atmosphere at night, *E*_n_ has also been reported across a range of taxa and biomes under various climatic conditions (Forster, 2014), certainly including the desert settings (Ludwig, Jewitt, & Donovan, 2006; Ogle et al., 2012; Snyder, Richards, & Donovan, 2003; Yu et al., 2016). *E*_n_ is often positively correlated with the nighttime leaf‐to‐air vapor pressure deficit (VPD) (Christman, Donovan, & Richards, 2009; Hogg & Hurdle, 1997; Pfautsch et al., 2011; Zeppel, Tissue, Taylor, Macinnis‐Ng, & Eamus, 2010; Zeppel et al., 2012) although some data indicate inverse relationship [see the review by Caird, Richards, and Donovan (2007)], or the combination of VPD and wind speed (Benyon, 1999; Phillips, Lewis, Logan, & Tissue, 2010), or the soil water availability (Cavender‐Bares, Sack, & Savage, 2007; Zeppel et al., 2012) and further regulated by the circadian clock (Resco de Dios et al., 2013). *E*_n_ may therefore result from increased stomatal conductance caused by a combination of high temperature, low humidity, and high soil water availability. These conditions are often typical to riparian forests in the hyperarid climate where soil water availability remains high because of trees’ access to shallow groundwater throughout the summer (Yu et al., 2016).

Nocturnal sap flow is commonly estimated using sap flow techniques (Burgess et al., 2001; Dawson et al., 2007; Zeppel et al., 2010); however, paritioning of noctunal sap flow into *E*_n_ and stem refilling (*R*_e_) components is methodologically difficult (Dawson et al., 2007). While *E*_n_ can potentially be observed directly via measurements of leaf‐level transpiration with canpoy or leaf chambers (Caird et al., 2007; Cirelli, Equiza, Lieffers, & Tyree, 2016; Ogle et al., 2012), a precise determination of *R*_e_ is more difficult. Several methods have been suggested to determine the *R*_e_ component of nocturnal sap flow. For example, Phillips et al. (2010) suggested an empirical method that is related to patterns of VPD across various days. Zeppel et al. (2010) estimated the contribution of nocturnal sap flow to *R*_e_ by subtracting the sap flow at the height of crown from that at the ground level. The “forecasted refilling” involves extrapolating forward the declining portion of the diurnal curve of sap flow until it approaches to zero flow as stomatal closure completely. The area above the extrapolated curve is identified as *E*_n_ and that below the curve is *R*_e_ (Fisher, Baldocchi, Misson, Dawson, & Goldstein, 2007). The “forecasted refilling” method has previously been applied to cloudless, rain‐free days and nights with low VPD (Alvarado‐Barrientos et al., 2014; Buckley, Turnbull, Pfautsch, & Adams, 2011).

The study site was located at a riparian forest dominated by *Populus euphratica* Oliv. trees (Euphrates poplar) and *Tamarix ramosissima* Ledeb. shrubs growing in a hyperarid climate in northwestern China. It provides an ideal system to observe HR and *E*_n_, given the relatively stable soil water availability due to the shallow water table and high evaporative conditions at night. Previously, HR was observed from *P. euphratica* and *T. ramosissima* roots (Yu et al., 2013), but the magnitude of HR in the *P. euphratica* trees was lower (Yu, Feng, Si, & Zhang, 2014) than that previously reported (Neumann & Cardon, 2012) and for which no immediate causal explanation was evident. Furthermore, we previously observed a significant nocturnal sap flow at the site (Si, Feng, Yu, & Zhao, 2015) of which 34% was attributed to *E*_n_ roughly estimated as the sum of sap flow from midnight to predawn (Yu et al., 2016).

Given that, this study focused on the relationship between HR and *E*_n_ and/or *R*_e_ based on the hypothesis that the magnitude of HR was limited by *E*_n_ and/or *R*_e_ (Bauerle et al., 2008; Caird et al., 2007; Dawson et al., 2007; Howard et al., 2009). Specifically, we evaluate the contributions of these different sources of sap flow in this species by (1) separating nocturnal sap flow into *E*_n_ and *R*_e_ and (2) determining the relationship between HR and *E*_n_ and/or *R*_e_ of *P. euphratica* through in situ measurement in a hyperarid climate in NW China.

## 2 MATERIALS AND METHODS

### 2.1 Research area and field site

The research area is situated at the northwest of the Badain Jaran Desert and is dissected by the Heihe River, the second inland river of China. The climate of the region is extremely arid with the average annual rainfall of 37.5 mm, of which greater than 75% falls between June and August. Pan evaporation measured by E‐601B pan is 2,226 mm p.a. (2002–2015) (Yu, Feng, Si, Zhang, & Zhao, 2017a). The field site located at the lower Heihe river basin (lat 42°01′N, long 100°21′E, 934 m AMSL) is composed of remnant populations of broad‐leaved *P. euphratica* tree (Yu et al., 2017a), with the stand density of 146 individuals ha^−1^, an average height of 11.17 m, and diameter at breast height (DBH) of 45.9 cm (Table 1). *P. euphratica* contributes the majority (~75%) of total basal area in the study site. Sapwood area (*A*_s_) of the stand was estimated from the allometric model between *A*_s_ and DBH, *y* = 1.2828 *x* ^1.4,223^ (*R* ^2^ = .87, *p *< .01, *N *= 59) in the whole study area according to Keyimu, Halik, and Kurban (2017), in which sapwood width was firstly measured by caliper rule by taking 5‐mm‐diameter cores (Haglof, Sweden), and then, *A*_s_ was estimated by combining it with DBH. Soils at the site are sandy loam and silt loam above 82 cm and below 124 cm, respectively, with a distinct sand layer between them. The soil moisture content was obtained gravimetrically from soil pits during the growing season of 2012 and then multiplied by soil bulk density to obtain soil volumetric moisture content (θ). Soil, *c*. 2 m west of the selected trees, was sampled monthly using an auger from 0 to 160 cm depth at 20‐cm intervals (three sets of samples were obtained). Vertical profile of θ showed a distinctly higher value at 20–60 cm with an average of 16.7% in stand, and it was in consistent with the fine root (<2 mm) length density that suggests potentially HR (Yu et al., 2013).

**Table 1:** Summary of biological parameters for three selected *Populus euphratica* trees and quadrat with the size of 100 m × 100 m for this study area

Trees	Height (m)	DBH (cm)	*A*_s_ (cm^2^)	*A*_c_ (10^3^ cm^2^)
Stem	11.1	43.35	268.68	220.51
	11.4	49.47	324.35	301.75
	12.1	41.38	251.49	255.05
Mean ± *SD*	11.53 ± 0.51	44.73 ± 4.22	281.51 ± 38.09	259.10 ± 40.77
Stand (*N* = 146)	11.17 ± 2.35	45.90 ± 14.38	304.48 ± 143.74	292.46 ± 142.92
Sig. (2‐tailed)	0.343	0.679	0.406	0.292

DBH, diameter at breast height; *A*_s_, sapwood area; *A*_c_, projected area of crown.

### 2.2 Meteorological data

Meteorological parameters were measured at a height of 3 m in a nearby clear space and included the following: net radiation (*R*_n_, W/m^2^), air temperature (*T*_a_, °C), relative humidity (RH, %), and wind speed (*U*, m/s). These data were recorded at 0.5‐hr intervals in 2012 (CR3000, Campbell Inc., USA) (Yu, Qi, Si, Zhang, & Zhao, 2017b). Daily rainfall (mm) was acquired from the nearest meteorological station belonging to the China Meteorological Administration and located in Ejin, which is 8 km from the study site. VPD was calculated from *T*_a_ and RH.

### 2.3 Sap flow measurements

Three mature trees were randomly selected to measure the sap flow of stems and roots using the heat ratio method (Burgess et al., 2001) during the growing season of 2012, from first foliation stage to leaf coloration stage (roughly from April 4th to October 28th) and after that until the foliation stage of the next year was classified as the dormancy period. The selected trees were representative of the surrounding stand and had similar height, DBH, and *A*_s_ with stand (one‐sample *t*‐test, *p* = .05) (Table 1). The sap flow probe sets used in this study (SFM1, ICT Inc., Armidale, Australia) contain one heater and two temperature probes, positioned upstream and downstream of the heater (35 mm long). Each temperature probe measures sap velocity at 7.5 and 22.5 mm distance from the tip of the probe. Probes were radially inserted into the xylem tissue of the stem (i.e., northern side, 130 cm in height) and two lateral roots (i.e., northern and southern sides, respectively, 30 cm in depth distance to trunk) for the selected trees. The diameter and *A*_s_ of lateral roots were 6.90 ± 1.27 cm and 152.04 ± 55.15 cm^2^, respectively (Yu et al., 2013). Heat pulse velocity was measured at 0.5‐hr intervals. All corrections related to the probe wounds and misalignments and calculation of sap velocity (*V*_s_, cm/hr) on a sapwood area basis were made according to Burgess et al. (2001).

**Figure 1:**
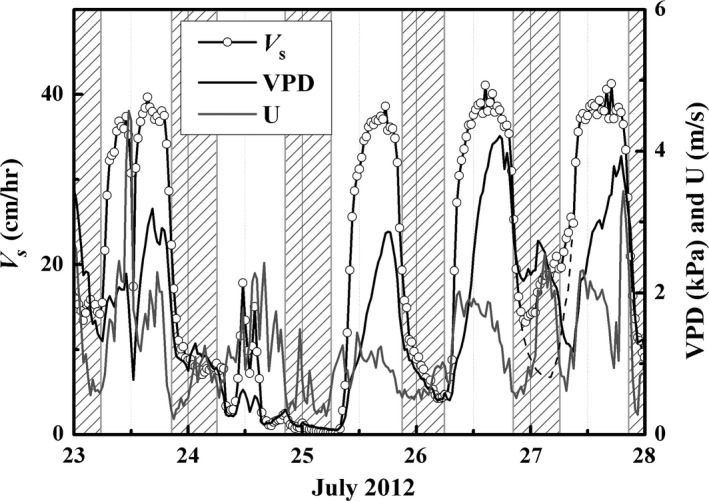
Hourly variation in stem sap velocity (*V*_s_, cm/hr) of *Populus euphratica*, vapor pressure deficit (VPD, kPa), and wind speed (U, m/s) that, in combination, drive nocturnal sap flow after a rainfall event (12.4 mm, 24th July). The shaded area represents the nighttime

Zero flow adjustment was of vital importance to determine *E*_n_ (Alvarado‐Barrientos et al., 2014; Zeppel et al., 2010). It can be determined using two methods: (1) severing the tree xylem to reduce the flow to zero and (2) estimating nocturnal *V*_s_ at night when atmospheric demand is low, that is, low VPD. Previous studies have shown that both methods have no significant differences (*p* < .05) (Zeppel et al., 2010). In this study, the cutting of the tree xylem was not possible because research sites were located in a natural forest national reserve. Instead, near‐zero sap flow was assumed during night (25 July 2012) with substantial rainfall (12.4 mm in 3 hr, Figure 1), causing VPD to be the lowest recorded throughout the entire measurement campaign (<0.18 kPa). Recorded *V*_s_ ceased quickly with the onset of rainfall and then stabilized at the lowest recorded sap velocities (1.6 cm/hr). To compare with dimension of HR, the sapwood‐related sap flow (*Q*_s_, kg/hr) was calculated by multiplying *V*_s_ by *A*_s_ and water density.

### 2.4 Separation of transpiration and refilling

Whole‐tree crown‐related sap flow (*Q*_c_, equal to transpiration *E*, mm/hr) was calculated by dividing the product *V*_s_ and *A*_s_ by the projected area of crown (*A*_c_, cm^2^). *A*_c_ was calculated as the circular area via measurement of diameter of crown at four directions. *Q*_c_ was separated into daytime (equal to *E*_d_, mm/hr), and nighttime *Q*_c_ according to *R*_n_ is greater than or less than 5 W/m^2^ (Daley & Phillips, 2006). Previously, Fisher et al. (2007) suggested that the early sloped phase of nighttime *Q*_c_ represents mostly *R*_e_ and the later, nonzero linear phase represents *E*_n_. Thus, we separated the *E*_n_ and *R*_e_ components of nighttime *Q*_c_ using an exponential decay model (*y* = a *e* ^b*x*^). The relationship between nighttime *Q*_c_ and VPD for the first 3 to 5 hr after dusk was determined with *R* ^2^ consistently >.97 (Figure S1), since nighttime *Q*_c_ from sunset to sunrise did not exponentially decreased (Alvarado‐Barrientos et al., 2014) but first decreased from sunset to midnight with decreased VPD and then stabilized and increased until sunrise of the next day, thus the above of the extrapolated curve is identified as *E*_n_ and below of the curve is *R*_e_. The last fitted *Q*_c_ data point was before an inflexion point was reached (i.e., when nighttime *Q*_c_ was not continuing to decline). Daily *E* (mm/day) was the 24‐hr sum of *E* from sunrise to sunrise of the next day, and then, the contribution of *E*_n_ to *E* (*E*_n_:*E*) was computed.

### 2.5 Hydraulic redistribution

HR was quantified as the total volume water estimated from negative sap flow (i.e., flow directionally away from the trunk) measured on roots. HR was presented as nighttime sap flow (kg/day), by summing the product of sap velocity by the cross‐sectional area of similar lateral roots and water density, instead of volumetric flow velocities (mm/day) owing to the latter it is difficult to scale up with the size of each individual lateral root monitored. Because it was difficult to measure sap flow on all roots, only large lateral roots with a west‐to‐east orientation were instrumented with sap flow sensors.

### 2.6 Statistical analysis

Mean and standard error (*SE*) of all variables were calculated. Difference between the mean of samples and population of stand (*N* = 146) was tested by the one‐sample *t*‐test at a significance level of α = 0.05. The differences in means of sap flow fluxes (*E*,* E*_d_, *E*_n_, *R*_e_) were examined via one‐way ANOVA at a significance level of α = 0.05 in conjunction with Tukey's post hoc test for the continuously measured data. The exponential decay modeling (*y* = a *e* ^b*x*^) was applied in the “forecasted refilling” method and to determine the relationship between sap flow fluxes (*E*_n_, *R*_e_, and *E*_d_) and HR. The sigmoidal function with three parameters (*f* = *a*/(1 + exp(−(*x* − *x*_0_)/*b*))) was used to determine the relationship between *E*_n_, *R*_e_, and *E*_d_. To determine the relationship between HR and *E*_n_, and *R*_e_, the stepwise regression analysis was used, which only considered the inclusion of additional parameters if an improvement of *R* ^2^ > 5% was shown and if parameter estimates were significant. Those statistical analyses and plotting were performed with the software package of SPSS Statistics (version 19.0; IBM, Armonk, NY, USA) and SigmaPlot (version 13.0; Systat Software, Erkrath, Germany), respectively.

## 3 RESULTS

### 3.1 Sap velocity and meteorological factors

Peak hourly *V*_s_ generally occurred in the early afternoon, whereas zero‐to‐low *V*_s_ occurred on nights of low VPD and high *V*_s_ occurred on nights of higher VPD and U. *V*_s_ reached a maximum velocity of ~ 40 cm/hr during the daytime. *V*_s_ was close to zero on the night of 24th/25th July when VPD was ~ 0 kPa following a 12.4‐mm rainfall event. On the night of 26th/27th July, when VPD was greater than 2 kPa and U greater than 1 m/s, nighttime *V*_s_ was relatively high (Figure 1).

**Figure 2:**
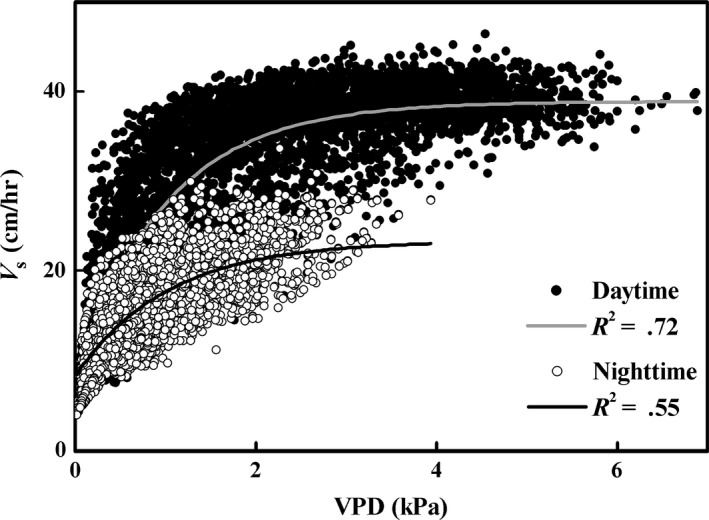
Relationship between sap velocity (*V*_s_, cm/hr) and vapor pressure deficit (VPD, kPa) throughout the growing season (from May 4th to October 15th) devoid of rain days in 2012 for *Populus euphratica*. Logarithmic relationships are shown for both daytime and nighttime

Compared to the daytime *V*_s_ with an average of 32 cm/hr, nighttime *V*_s_ averaged 16 cm/hr. When devoid of rainfall days, there was a significant (*p* < .01) logarithmic relationship between *V*_s_ and VPD for both daytime and nighttime periods during the growing season. However, the goodness of fit was higher in the daytime (*R* ^2^ = .72) than in the nighttime (*R* ^2^ = .55) (Figure 2).

### 3.2 Partitioning of nocturnal transpiration and xylem refilling

**Figure 3:**
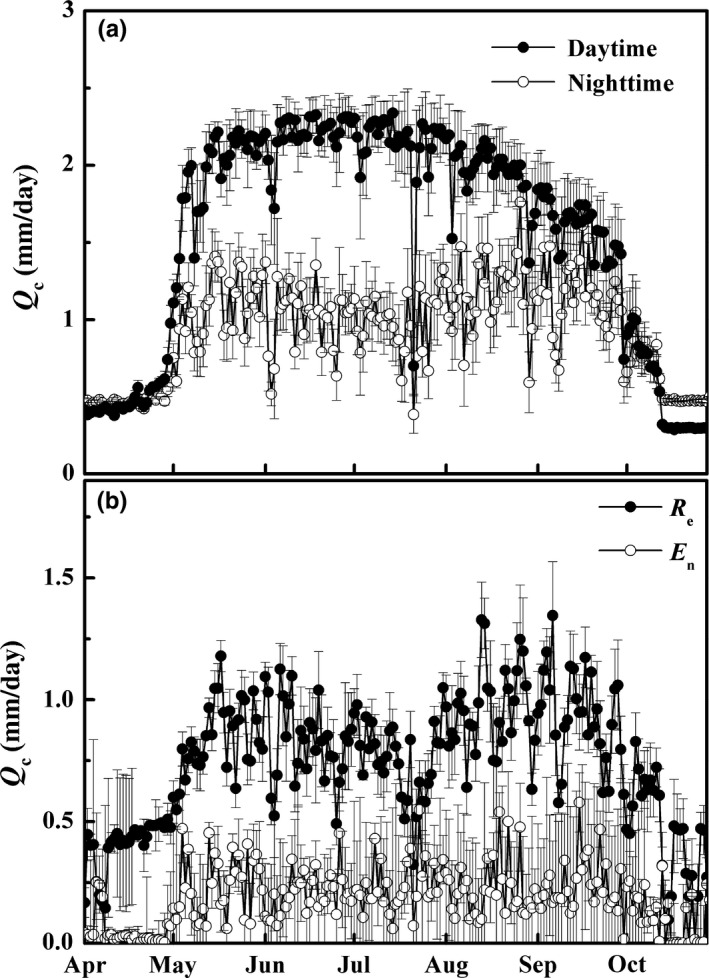
The daily variation (mean ± *SE*) in (a) sap flow (*Q*_c_, mm/day) at daytime and nighttime and (b) partitioned nocturnal transpiration (*E*_n_, mm/day) and stem refilling (*R*_e_, mm/day) during the growing season of 2012

Daytime *Q*_c_ increased suddenly during early May and slightly raised from May to mid‐July, and after that, it sharply decreased to October and maintained the lowest from mid‐October to the dormant period (Figure 3a). Substantial nighttime *Q*_c_ (38% of 24‐hr flux on average) was observed and is higher than daytime *Q*_c_ in defoliation period, but it is less than and opposite during daytime *Q*_c_ at the whole‐leaf stage (Figure 3a). Overall, the forecasted refilling method found that *R*_e_ and *E*_n_ on average accounted for approximately 80% and 20% of nighttime *Q*_c_, respectively. *E*_n_ was increased during early May and stabilized at the whole‐leaf stage and decreased after defoliation. *R*_e_, however, showed peaks in spring and autumn and a relative decline in mid‐summer, which is obviously higher than *E*_n_ (Figure 3b).

The monthly totals of *E*,* E*_d_, *E*_n_, and *R*_e_ and the contribution of *E*_n_ and *R*_e_ to *E* are presented in Table 2, in which *R*_e_ increased proportionally with *E*_d_, but not the case for *E*_n_. Compared to *E*_n_ with a mean of 7.5% of daily *E*,* R*_e_ was substantial, accounting for an average of 29.7% of daily, which suggests that high nighttime *Q*_c_ was mainly attributed to *R*_e_. *E*_n_ was consistent throughout the year with the *E*_n_:*E* ratio approximately 5% to 10%.

**Table 2:** Seasonal variation in daily transpiration (*E*, mm), daytime transpiration (*E*_d_, mm), nighttime transpiration (*E*_n_, mm), tissue refilling (*R*_e_, mm), and the contribution of *E*_n_ and *R*_e_ to *E* (*E*_n_:*E* and *R*_e_:*E*, %) during the growing season from April to October of 2012 for *Populus euphratica* trees

Months	4	5	6	7	8	9	10	Sum or mean
*E*	24.2	87.5	96.7	96.6	99.7	83.3	36.2	524.3
*E*_d_	12.4	56.0	65.5	66.3	62.5	48.2	18.1	329.0
*E*_n_	1.2	6.6	6.5	7.2	7.3	7.2	3.5	39.4
*R*_e_	10.6	24.9	24.7	23.2	29.9	27.8	14.7	155.9
*E*_n_:*E*	5.0	7.6	6.7	7.4	7.3	8.7	9.6	7.5
*R*_e_:*E*	43.9	28.4	25.6	24.0	30.0	33.4	40.6	29.7

**Figure 4:**
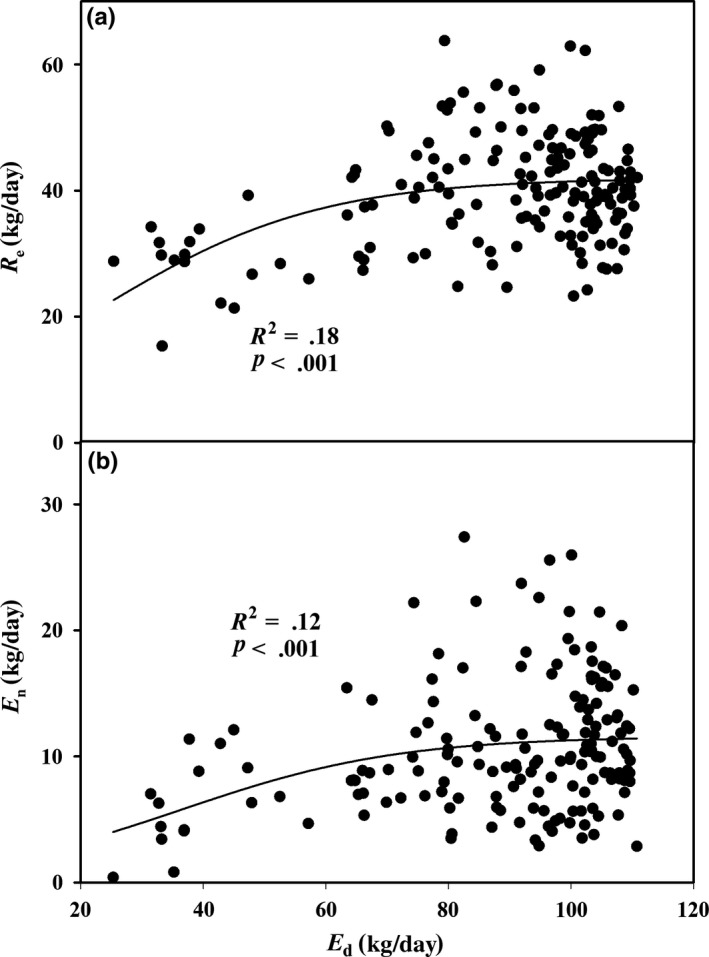
Relationships between daytime transpiration (*E*_d_, kg/day) and (a) stem refilling (*R*_e_, kg/day) and (b) nighttime transpiration (*E*_n_, kg/day) during the growing season of 2012. Sigmoidal function with three parameters (*f* = *a*/(1 + exp(−(*x* − *x*_0_)/*b*))) is used to fit the relationships

A significant logarithmic relationship was observed between *E*_d_ versus *E*_n_ and versus *R*_e_, but the goodness of fit was better for the latter (*R* ^2^ = .18) than for the former (*R* ^2^ = .12) (Figure 4) throughout the growing season (when days of rainfall were removed from the analysis).

### 3.3 Relationship between hydraulic redistribution and nocturnal transpiration and stem refilling

**Figure 5:**
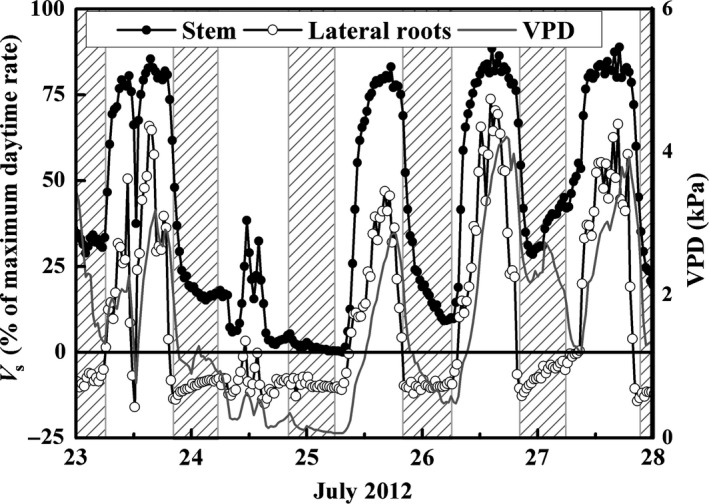
Hourly variation in sap velocity (*V*_s_, as a percentage of maximum daytime rate, %) of stem and lateral roots of *Populus euphratica* and vapor pressure deficit (VPD, kPa) over five successive days surrounding a rainfall event (12.4 mm, 24th July) of 2012. The shaded area represents the nighttime

**Figure 6:**
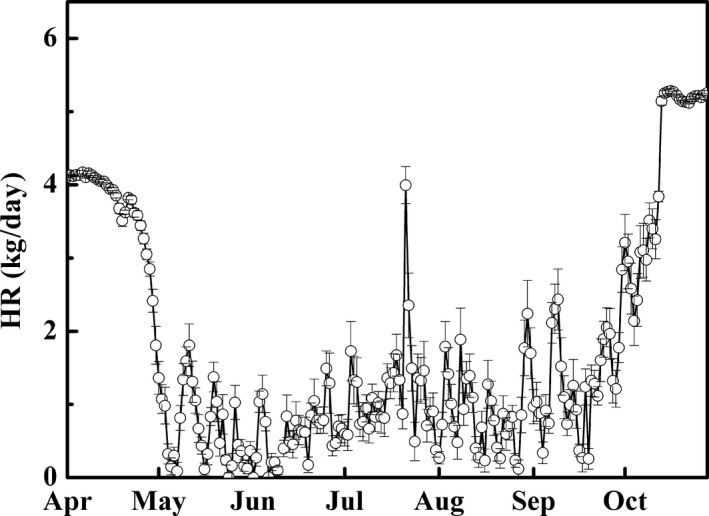
The daily variation (mean ± *SE*) in hydraulic redistribution (HR, kg/day) of roots of *Populus euphratica* during the growing season of 2012

Devoid of the rainfall days, *V*_s_ of stem was always positive; conversely, *V*_s_ in lateral roots was positive at daytime but negative at nighttime, that is, moving away from the base of the stem toward the root tips, which strongly suggests HR. Comparisons between *V*_s_ (as a percentage of maximum daytime rate, %) of stem and lateral roots show variation (Figure 5), whereby reverse nighttime *V*_s_ increased in lateral roots after rain (25th and 26th July) and rapidly decreased with rising daytime *V*_s_ later in the second day (27th July), which suggests that HR was possibly limited by sap flow of stem, *R*_e_ and/or *E*_n_. Daily HR of roots (Figure 6) was reverse to the variation of *Q*_c_, in particular to the daytime *Q*_c_ (Figure 3).

**Figure 7:**
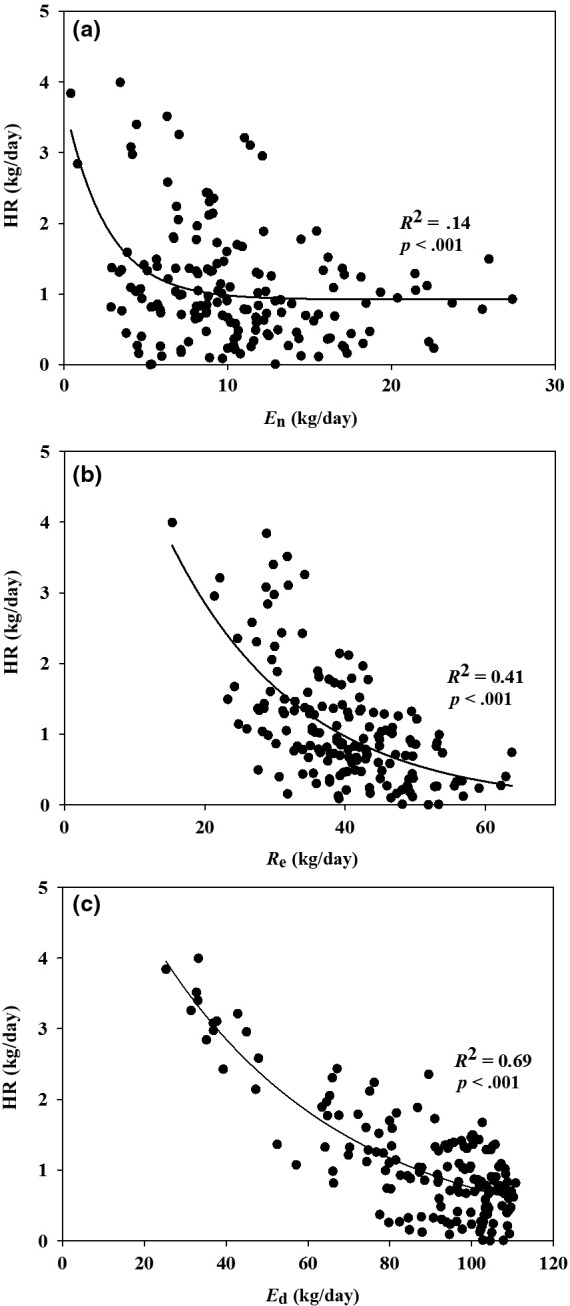
Relationships between hydraulic redistribution (HR, kg/day) and (a) nighttime transpiration (*E*_n_, kg/day), (b) stem refilling (*R*_e_, kg/day), and (c) daytime transpiration (*E*_d_, kg/day) during the growing season of 2012. Exponential decay function (*y *= *a* exp(−b*x*)) is used to fit the relationships

A significantly exponential decay relationship was found between HR and *E*_n_ (Figure 7a) and *R*_e_ (Figure 7b) at daily scale during the growing season, and the goodness of fit was better for *R*_e_ (*R* ^2^ = .41) than for *E*_n_ (*R* ^2^ = .14), which suggests that HR was mainly inhibited by *R*_e_ and *E*_n_. A negative coefficient of multiple linear regression model (HR = −0.533*R*_e_ − 1.522*E*_n_ + 3.563, *R* ^2^ = .41, *p* < .001) and mixed exponential model (HR = 2.051exp (−21.682*R*_e_) + 7.719 exp (−2.559*E*_n_), *R* ^2^ = .47, *p* < .001) also demonstrated the decreased HR with the increasing *R*_e_ and *E*_n_. A stepwise regression analysis showed that the proportion of the variance in the model explained by *R*_e_ and *E*_n_ (*R* ^2^ = .40) was 4% higher than that explained by *R*_e_ alone (*R* ^2^ = .36, *p* < .001). And, it is interesting that HR was significantly decreased with the increasing *E*_d_ (*R* ^2^ = .69) (Figure 7c).

## 4 DISCUSSION

### 4.1 Substantial nocturnal sap flow: transpiration or stem refilling?

Nocturnal water loss was once thought to be zero, or negligible; however, over the last two decades, a growing body of researchers have observed that it can contribute significantly to total daily water use across a range of natural ecosystems (see the review by Forster, 2014). The significance of nighttime *Q*_c_ to daily sap flux has been defined in a number of ways and one of which as a proportion of whole‐day flow has been reported as high as 38% and 39% in *Phillyrea latifolia* and *Quercus ilex*, respectively (Barbeta, Ogaya, & Peñuelas, 2012). Particularly, the ratio of nighttime sap flow in the C_3_‐CAM species, *Clusia minor*, can be up to 70% with the increase in nocturnal acid accumulation (Herrera, Ballestrini, & Tezara, 2008). For *P. euphratica*, the ratio of nighttime *Q*_c_ to total daily flow (38%) was significantly higher than the average of 12% across seasons, biomes, and phylogenetic groups (Forster, 2014). Nocturnal *Q*_c_ ratio in other poplar species has an average of 7.71% and 9.33% with a maximum record of 29.07% and 23.93% for *Populus grandidentata* and *Populus deltoides*, respectively (Marks & Lechowicz, 2007), and continental (i.e., arid) biomes have an average of 13.88% with a maximum record of 24.37% using the sap flow measurement (Forster, 2014), all of those less than those reported in this study. However, Rohula, Kupper, Räim, Sellin, and Sõber (2014) using weighed method of excised shoots growing in the flasks of greenhouse found high nighttime water loss (i.e., >50%) for *Populus balsamifera* L. and *Populus* × *wettsteinii* Hamet‐Ahti just at predawn. Therefore, our studied species growing in a natural environment with a hyperarid climate had an exceptional rate of nighttime *Q*_c_, which is a significant proportion of total daily *Q*_c_ with observed values on some nights over 50%.

However, the relative contribution to nighttime *Q*_c_ via either *E*_n_ or *R*_e_ is unknown largely due to methodological difficulties in accurately assigning nighttime *Q*_c_ to *E*_n_ or *R*_e_ (Zeppel, Lewis, Phillips, & Tissue, 2014). Just as Fisher et al. (2007) stated, Resco de Dios et al. (2013) also attributed the initial decrease in *Q*_c_ early in the night (the first *c*. 3 hr after dusk) to a sluggish stomatal response and/or stem water refilling. Similarly, the initial decrease time owing to stem refilling is up to 5 hr after dusk in *Eucalyptus saligna* growing in whole‐tree chambers (Zeppel et al., 2011). Consistent with these studies, we also thought that the initial decrease in *Q*_c_ after dusk was primarily induced by the stem refilling for the daytime water loss (Figure S1).

While *E*_n_ has been observed across a range of species, plant function types, and environments (Benyon, 1999; Caird et al., 2007; Dawson et al., 2007; Ogle et al., 2012; Snyder et al., 2003), its regulating mechanisms remain poorly understood (Zeppel et al., 2014). With the exception of the exogenous environmental regulation, for example, soil moisture content, VPD, and U (Zeppel et al., 2014), recent studies demonstrated that circadian clock also plays an important role in regulating nighttime water use (Resco de Dios et al., 2013), in which the contribution of circadian regulation to nocturnal *Q*_c_ variation (23%–56%) was comparable to that of VPD (25%–58%). This study shows that VPD can account for 55% of variation in nighttime *V*_s_ (Figure 2), indicating the potentially combined effect of exogenous and endogenous regulation on nighttime *Q*_c_, which was also supported by the rising *Q*_c_ with the decreased VPD at predawn (Figure S1. e). Therefore, we concluded that the substantial nighttime *Q*_c_ was constituted by water loss from leaves via stoma (i.e., *E*_n_) and water refilling of tree capacitance to replace water lost via daytime transpiration (i.e., *R*_e_).

It is notable that proportions of *E*_n_/*E* were lower with an average of 7.5% for *P. euphratica* (Table 2) than those of the semiarid region of the western United States (10%–32%) (Snyder et al., 2003), northwestern Australia (reached a maximum of 50%) (Pfautsch et al., 2011), and a Mediterranean holm oak forest (10%–30%) (Barbeta et al., 2012). However, *R*_e_ was significantly higher, accounting for 80% of nighttime *Q*_s_, than that of two evergreen temperate woodland species (30%–50%, Zeppel et al., 2010), but was comparable to that of Mediterranean trees and shrubs (Fisher et al., 2007) and a mixed New England deciduous forest (Daley & Phillips, 2006). In addition to the uncertainty of “forecasted refilling” method (Alvarado‐Barrientos et al., 2014; Buckley et al., 2011), the high *R*_e_ potentially resulted from large stem water capacitance of studied species (observed the spouting sap out hole after drilling the tree‐cores), that enables the trees to maintain maximum or near maximum transpiration rates for a longer period of time (Figure 3), just as in the tropical forest canopy trees, *Anacardium excelsum* and *Ficus insipida* (Goldstein et al., 1998). That *R*_e_ can contribute one‐third of total *E* (29.7%) is a unique result, and then, the high internal water storage capacity may provide insights into tree species’ water relations and water use strategies in extremely dry desert riparian ecosystems.

### 4.2 Limited hydraulic redistribution: nocturnal transpiration or stem refilling

Based on the whole‐tree water balance theory, the nighttime root water uptake was the sum of HR, *E*_n_, and the refilling of internal water reservoirs (roots, stem, and leaves, i.e., *R*_e_) (Dawson et al., 2007), which indicated that HR may be depressed by the large fluxes of water moving toward the canopy in the stem (*R*_e_ and *E*_n_). Consistent with this theory, greenhouse studies have shown that HR is experimentally reduced when *E*_n_ is increased by lighting at night (Bauerle et al., 2008; Caldwell, 1990; Caldwell & Richard, 1989). Howard et al. (2009) demonstrated that HR can also increase by artificially suppressing *E*_n_ via canopy bagging of *Artemisia tridentata* and *Helianthus anomalus* subjects, which is similar to Scholz et al. (2008).

Although the “forecasted refilling” method (Fisher et al., 2007) was previously applied to cloudless, rain‐free days and nights with low VPD (Alvarado‐Barrientos et al., 2014; Buckley et al., 2011), we suggest that the method could be applicable to fit the decreased *Q*_c_ owing to *R*_e_ under moderate VPD in night with potentially overestimated but less effect on the relationship between *R*_e_ and HR. Indeed, we observed that the leaf stomatal conductance and transpiration rate were close or equal to zero from sunset to midnight across different seasons (Yu et al., 2016). The negative logarithmic relationship between HR and *R*_e_ was better than *E*_n_ (Figure 7), which suggested that HR is predominantly controlled by *R*_e_ and subsequently by *E*_n_. It is demonstrated that the more water loss in daytime, the more water recharge in nighttime (Figure 4) and then less water redistribution to the soil layer via roots (Figure 6), which could explain the stronger relationship between HR versus *E*_d_ and *R*_e_ than between HR versus *E*_n_ (Figure 7). Under the stable resistance of sapwood, the root water uptake was preferentially transferred to the stem and then canopy to recharge water lost (Figure 5) rather than roots (Figure 6). Therefore, HR was depressed by the upward sap flow draw via refilling of unsaturated tissue and then transpiration, both of which acted as competing water sink.

The magnitude of *R*_e_ indicates the higher capacitance of stem, which is a measurement of the store water of plants (McCulloh, Johnson, Meinzer, & Woodruff, 2014) that could buffer the system by reducing the xylem pressure drop when transpiration increases (Meinzer, Domec, Johnson, McCulloh, & Woodruff, 2013). Studies found that stem water storage was negatively associated with wood density in five coexisting temperate broad‐leaved tree species (Köcher, Horna, & Leuschner, 2013), suggesting the higher water storage as lower wood density. Many studies suggest that most *Populus* species are highly vulnerable to cavitation causing 50% loss of hydraulic conductivity (*P*_50_) occurring between −1 and −2.5 MPa (Hacke, 2015; Hukin, Cochard, Dreyer, Le Thiec, & Bogeat‐Triboulot, 2005; Pan, Chen, Chen, Wang, & Ren, 2016). For *P. euphratica*, the basic wood density of stem was less than that of the co‐occurring species, *T. ramosissima*, 0.41 versus 0.73 g/cm^3^ (Yu et al., 2013), suggesting that *P. euphratica* can store more water than *T. ramosissima*. Although the nocturnal water fluxes may be a key process driving refilling of storage of sapwood (Daley & Phillips, 2006) and HR (Bleby, McElrone, & Jackson, 2010), this remains generally untested for *Populus* species. Stem refilling is crucial for trees to avoid xylem cavitation and water deficit (Daley & Phillips, 2006; Zeppel et al., 2014) and need further investigation particularly for the riparian trees growing in the hyperarid area.

Our study also demonstrated that *E*_n_ can reduce the magnitude of HR, a finding that may have further implications for arid riparian trees under the future climate where nighttime temperatures are projected to rise at higher rates than daytime temperatures (Peng et al., 2013). This relationship may have important disadvantageous consequences for plant survival in arid area. Experimental evidence and the use of novel modeling approaches suggest that HR may affect tree water use and productivity at the community scale (Domec et al., 2010; Prieto et al., 2012). Models that incorporate HR but not consider *E*_n_ into an atmospheric general circulation model may overestimate the impact of HR on ecosystem water use (Jackson, Sperry, & Dawson, 2000) and seasonal climate cycles (Lee, Oliveira, Dawson, & Fung, 2005), and it therefore should be considered based on the negative effect of *E*_n_ on HR in the future climate change scenarios.

## 5 CONCLUSION

Substantial nighttime *V*_s_ was observed and increased with VPD but with low determination coefficient (*R* ^2^ = .55), which suggests that the nocturnal *V*_s_ should be attributed to both of *E*_n_ and *R*_e_. We estimated that *E*_n_ accounted for approximately 7.5% of 24‐hr sap flow, compared to *R*_e_ of 29.7%. Here, we showed that the magnitude of HR can partially reduce by naturally occurring *E*_n_, which would increase under further climate change scenarios. Surprisingly, the negative exponential decay relationship between HR and *R*_e_ was better than *E*_n_, which demonstrated that HR of roots was depressed more by stem refilling than by nocturnal transpiration for *P. euphratica* Oliv. through in situ measurement in a hyperarid climate in NW China.

## CONFLICT OF INTEREST

We declare that we have no conflict of interest.

## AUTHOR CONTRIBUTION

T. Y. and C. Z. carried out the experiment and analyzed the data and wrote the first draft. Q. F., J. S., and X. Z. obtained the funds to support the project and reviewed the article. P. M. and M. F. reviewed the article and helped to revise the language.
